# On Fallacies in Neuroscience

**DOI:** 10.1523/ENEURO.0491-20.2020

**Published:** 2020-12-10

**Authors:** Christophe Bernard

A fallacious argument is one that seems to be valid but is not so. Why are fallacies so commonplace in scientific papers, and why can we not detect them when we read them? This editorial attempts to address these questions, which are central to do better science.

As a working example, let us consider a paper I read in a high-profile journal, which caught my attention. The title was “whoa,” potentially a ground-breaking finding. I read the abstract, in which the authors describe their results: Following a given brain insult, there is cell death. When gene *x* is removed, cell death is considerably increased. The conclusion of the abstract and title reads: “protein X is neuroprotective.” A red light started to flash in my brain. I thought that, maybe, the authors could not describe all their results in the abstract. After reading the results section, I found that all they had done was to compare cell death following an insult in the presence and absence of X. Yet, the conclusion was “X is neuroprotective.” The latter is a very strong statement, as it implies that we now have a cool target to prevent cell death. However, the paper does not contain any data supporting this conclusion. I then asked myself: how come the reviewers did not notice it? What about the handling editor? Of course, this makes a very sexy title, one that attracts readers.

I decided to run an experiment. I contacted several scientists, including editors, and asked them to read the paper and tell me what they thought about it. The answer was “great story.” After asking them to read the title and abstract carefully, still, nothing wrong was detected. I did the same at my institute. Only one PhD student detected it.

Why can we not see it? The answer may not be that simple, and I am convinced that even the authors were unaware of it. I thought I could see the fallacy because I was trained in mathematics, hence formal logic. Philip Johnson-Laird and colleagues argue that the basis of human reasoning is not formal logic (Johnson-Laird, 2010). When I asked the PhD student what she detected, she told me that “the conclusion is not correct because the authors did not consider other possibilities when interpreting the data.” In a nutshell, this is the take-home message: Ignoring other possibilities or interpretations may be the main source of fallacies.

Many things have been written on fallacies. If you are curious about it, past and current ideas can be found here: https://plato.stanford.edu/entries/fallacies/. It all started with the identification of 13 fallacies by Aristotle listed in *Σοφιστικοὶ Ἔλεγχοι* (https://plato.stanford.edu/entries/fallacies/#Ari). Another key reading on reasoning is Immanuel Kant’s *Kritik der reinen Vernunft* (Kant, 1889), but it is tough to read (I recommend reading interpretations of Kant’s thought). Accessible answers can be found in a more recent book, a milestone published in 1970: Charles Hamblin’s *Fallacies* (Hamblin, 1970). A field developed henceforth, closely linked to the theory of argumentation. Ramifications can be found in daily life, including politics and social media, which exemplify the constant use of fallacious arguments, sometimes leading to dramatic consequences.

Training in critical thinking and informal logic may help us not to create fallacies and to detect them. Unfortunately, this is not part of the usual neuroscience course. One solution I found is to tell trainees to ask themselves whether the conclusion is supported by the data, whether other interpretations can be proposed, etc., which is also the basis of the review process. This may not be as easy as it sounds since when we are reading a paper, we tend to follow the reasoning and logic of the authors, and if the argumentation is nicely laid out, it is difficult to pause, take a step back, and try to get an overall picture (see below). The same process may occur when writing a paper; my papers contain fallacies.

To try to understand the underpinnings of fallacies in neuroscience, it is useful to start with some common ones.

## Two Examples of Fallacies

I already described fallacies in studies using optogenetics (Bernard, 2020a) and field potential recordings (Bernard, 2017). Let us consider a classic example from fMRI studies: if I experience fear, then region Z lights up in my brain. Two conclusions are usually derived: if region Z is activated, I am experiencing fear and region Z is processing fear information. The first conclusion is the result of what is an example of reverse inference in the field. The fallacy is “to infer the converse from a conditional.” However, a relation is not necessarily convertible if the terms cannot be validly interchanged. Said differently, “if I am experiencing fear, then Z is active” does not imply that “if Z is active, then I am experiencing fear.” A more careful approach should have led us to test other paradigms. We do it, and one paradigm reveals that the sound of a kid laughing can also activate Z. This result does not refute the proposal (or conditional) that “if fear then Z is active,” but it refutes the proposal “if Z is active then fear,” because we just found that both fear and the sound of a kid laughing activate Z. As mentioned above, not considering alternative possibilities leads to fallacies.

Let us now consider the fallacy of “affirming the consequent.” If I am writing: “When I am reading Shakespeare in the MRI scanner, there is a strong bold response in area tempestas. Thus, area tempestas codes for Shakespeare books.” You would start to react and think, hmm, I am not sure about this proposal. But if I am now writing the classic example: “If Bacon wrote Hamlet, then Bacon was a great writer. Bacon was a great writer. Therefore, Bacon wrote Hamlet.” You would not think twice and immediately detect the fallacy. Yet, it is the same reasoning as for the region Z and fear example. Why is it difficult to detect the first fallacy (Z codes for fear) and not the third one (Bacon wrote Hamlet)? Perhaps, because the first one is plausible, while the third one immediately triggers an error signal since you know that Bacon did not write Hamlet.

In the case of the neuroscience example, the correct proposal would be that region Z *may* be involved in fear processing. The relation is perhaps convertible, but more experiments are needed. It is an interesting hypothesis, though.

Another classic fallacy is the fallacy of “denying the antecedent.” The paper mentioned at the beginning is a good example: “If I remove protein X, there is massive cell death in a pathologic context. Thus, protein X prevents massive cell death in a pathologic context.” Most of us do not detect it. But if I write it like this, “If I remove Shakespeare from all libraries, there is massive loss in culture. Thus, Shakespeare prevents a massive loss of culture.” You identify the fallacy immediately. Again, “X *may* prevent cell death” would have been the correct formulation (as a perspective at the end of the discussion, but not as a title); we do not know yet, but it is an interesting hypothesis to test.

## Why Does It Happen at All?

To some extent, humans can reason in terms of pure logic, like in mathematics, when we demonstrate a proposal or solve a problem. Rules are strict, and the result will be correct or not, and everyone sufficiently trained will agree.

Experimental sciences like neuroscience rely on interpretations of observations in a total absence of a framework. Hence, we cannot refer to a “truth.” In contrast, Euclidian geometry relies on five axioms, from which you can derive a deductive and logical system. In everyday life, Euclidian geometry is enough. In neuroscience, we do not know how the brain works, and we do not even have a theory of brain function or even the equivalence of axioms on which to build a deductive system.

An entry point to understand why fallacies are so common and not detected is the theory of “mental models and human reasoning” (Johnson-Laird, 2010). In the conclusion, the author writes: “Human reasoning is not simple, neat, and impeccable. It is not akin to a proof in logic. Instead, it draws no clear distinction between deduction, induction, and abduction, because it tends to exploit what we know. Reasoning is more a simulation of the world fleshed out with all our relevant knowledge than a formal manipulation of the logical skeletons of sentences. We build mental models, which represent distinct possibilities, or that unfold in time in a kinematic sequence, and we base our conclusions on them.” It has also been argued that human reasoning is parallel, rather than sequential, and that emotions can play an important role in the inferences we can make, particularly motivated inference (Thagard, 2011). Going back to the first example, “cell death” is highly emotionally charged, we do not want it to happen in our brain, right? Hence, when writing or reading about cell death motivated inference may play a role subconsciously.

Readers can also refer to my editorial on the role of “naming” in science, in particular the use of emotionally charged words (Bernard, 2020b). It is possible that when we write and read science, the fast and slow modes of thought are constantly interacting with each other. The fast system is instinctive and emotional, while the slow system is more deliberative and logical, involving different brain areas (Kahneman, 2011). Thus, emotionally charged words may bias our reasoning because they would be processed first.

Human reasoning can be seen as an emergent property of a complex system (the brain), characterized by complex nonlinear dynamics driven by multiple factors, the opposite of a linear proof in logic. During my exchanges with Philip Johnson-Laird, he argued that “the bottleneck for human thinking is the processing capacity of working memory, that is, its ability to handle the results of intermediate computations,” which would limit our ability to keep in mind alternate possibilities while thinking/writing/reading. To identify fallacies, we would need to pause and reflect on these possibilities. To quote him again: “One consequence is that people see causation when it is not there: magical thinking as the anthropologists refer to it.” The take-home message is that we need to accept human thinking limitations and not point an accusing finger to those drawing fallacious conclusions. Most of the time, it is not done consciously. This is the way we build a model of the world and think the way we think. It is bound to be imperfect (looping back to Kant).

## Can We Improve Things?

As for everything, education is the key. It is important to maintain a critical eye. Do facts support my conclusions? As mentioned in the preceding paragraph, the task may not be that easy. Contrary to pure logic, many factors come into play. It is important to consider mental products and emotions when engaged in critical thinking. I consider that developing such skills is the condition sine qua non before doing science. Why? Because fallacies prevent us from moving forward. To end up with a concrete example, I recently reviewed a paper in which the hypothesis was based on “we know that neuron N controls function F.” Several papers were cited as a justification. Checking all of them, I discovered that they only showed that when you disrupt N, you affect F. But they were all concluding that N controls F. There was no single piece of evidence that N controls F (i.e., in the absence of perturbation). It *may* be true. Note that this reasoning is inherent to most of our brain function approaches: we need to perturb the system. The illusion of causality has been present as soon as we started to perform lesions to investigate function. Manipulating the genome (KO and KI animals), optogenetics, chemogenetics, etc. proceed with the same idea: perturbing X results in Y. It does not show that X controls Y, but it is consistent with the latter proposal (Bernard, 2020a). This illusion of causality allows us to make progress, and most often than not, an abductive argument may be true in the end (even if it is logically wrong stricto sensu, which made me react when reading the paper about protein X). The real danger is to transform something plausible into a truth. This is how dogma emerges, and it is dangerous because it constrains true hypothesis testing and the reporting of non-supportive data. Abductive arguments can accelerate knowledge and, at the same time, slow down our path toward a better understanding of how the brain works if we are not careful enough.

I think that fallacies are inherent to human reasoning, as we may need them to make sense of the unknown. If we were to train students to detect fallacies systematically, they would become overly critical. This would impede the acquisition of critical thinking needed in science. I argue that good reasoning skills can still be taught and acquired if we always keep in mind the limitations of human reasoning and its apparent facility for fallacies. In practice, the first question we should ask is: are there other interpretations?

I cannot find a better way to summarize what I think than quoting Claude Bernard (Bernard, 1865): “Even when attempting to verify one’s inference by an experiment or an observation, it is necessary to remain the slave of the observation, as well as of the experiment. One must not be overcome by one’s inductive idea that is nothing but a hypothesis. I can say that I follow such a precept. Thus, the verification of my inferring hypothesis, whatever its likelihood, does not blind me. I hold conditionally to it. Therefore, I am trying as much to invalidate as to verify my hypothesis. In short, I do research with an open mind. This is the reason why I so often found results I was not looking for while investigating other things I could not find. The truth must be the goal of our studies. Being satisfied by plausibility or likelihood is the true pitfall.”

After all, the Tempest may still be located in area tempestas and Bacon may have written Hamlet.

I wish to acknowledge the nourishing discussions I had with Alexandre Billon, Philip Johnson-Laird, and Shahid Rahman.
